# A Case of Suppurating Middle Ear, Thrombosis and Phlebitis of Lateral Sinus, Death

**Published:** 1887-03

**Authors:** W. H. Harsant

**Affiliations:** Surgeon to the Bristol Royal Infirmary


### A CASE OF SUPPURATING MIDDLE EAR, THROMBOSIS AND PHLEBITIS OF LATERAL SINUS, DEATH. By W. H. Harsant, F.R.C.S., Surgeon to the Bristol Royal Infirmary.

It is a well known fact that suppuration of the middle ear may terminate fatally in two ways: 1st, by purulent meningitis, or by the formation of cerebral or cerebellar abscesses; and 2nd, by septic phlebitis, thrombosis, and septicæmia.

In former times, when both these conditions were looked upon as hopeless ones, it was a matter of comparatively little importance to diagnose between them; but now that it has been so well proved that cerebral abscesses may be treated with great hope of success (two successful cases having been reported lately—one by Dr. Gowers and Mr. Barker, in the *British Medical Journal* of Dec. 11th, 1886; and the other by Professor Greenfield, in the same *Journal* of Feb. 12th, 1887), it becomes a matter of the greatest importance to distinguish between localised collections of pus in the cranium, on the one hand and thrombosis and phlebitis of the cerebral sinuses on the other. It has been laid down by Politzer that the symptoms of these two conditions are quite distinct, and that the differential diagnosis ought to be at all times easy. He says: "There is danger of confounding thrombosis of the lateral sinus with meningitis and cerebral abscess only in the case of the simultaneous appearance of the sinus affection with these brain diseases. When such is not the case, the differential diagnosis is all the easier, as the groups of symptoms characterising both forms of disease present many divergencies. Whilst in meningitis and cerebral abscess there are neither such severe rigors nor such high temperatures as in sinus-thrombosis, *in the latter we find the brain symptoms only slightly marked, consciousness often remaining unimpaired even up to the end.*" *(Text-book of Diseases of the Ear.)*

Hartmann says: "As distinguished from the formation of cerebral abscess, the most important and earliest symptom of phlebitis is severe rigors. The pain is very great, limited to the region of the inflamed sinus wall, and increased on pressure. *Great restlessness sets in early, with delirium, convulsions, and extreme prostration.* Sometimes a temporary remission of the symptoms occurs, but the patient sinks, and coma and death ensue." *(Diseases of the Ear and their Treatment.)*

It may be seen, therefore, that two of the greatest living authorities on ear diseases give totally different accounts of the symptoms observed in phlebitis of the lateral sinus set up by ear disease, and further records of cases are required to enable us to distinguish accurately between this condition and cerebral abscess.

*Case.*—A shoemaker, aged 17, was admitted into the Infirmary, under my care, on July 20th, 1886. He had suffered from a purulent discharge and deafness in both ears since an attack of measles in early life. The left ear had given him attacks of pain from time to time, and the deafness in this ear had been greater than in the other. Two years before admission he had suffered from a severe attack of swelling and pain around the left ear, which had made him very ill for some weeks; but after free discharges from the meatus he recovered from this, and remained in his usual health until four days before admission, when he was taken with severe pain in the left side of the head and in the left ear: he noticed also an increase in the purulent discharge from the meatus. On the day before admission he had a fit, and vomited.

*On admission* he was in a dull and heavy condition, sleeping constantly, coiled up on the right side. He could be roused with difficulty, and then answered questions in a morose and irritable manner; he yawned frequently, and expressed a constant desire to be left alone and allowed to sleep. The left meatus was filled with a polypus, and a profuse fœtid purulent discharge exuded from it. There was a slight purulent discharge from the right meatus also; but owing to the severity of his condition, this ear was not examined. His temperature was 104°, pulse 96; and he refused all food except small quantities of milk.

I thoroughly syringed out the left meatus with warm boracic lotion, removing by this means a quantity of inspissated secretion which was pent up in the tympanic cavity behind the polypus; I then insufflated powdered iodoform into the meatus.

On the next day he was decidedly worse: there was rigidity of the right arm and leg, and I was told that during the night he had had several severe rigors. His temperature was 106.5°, pulse 100, and he lay in a semi-comatose condition, from which he could not be roused. There was no vomiting, but he had several rigors during the day.

On the following day, after a consultation with my colleagues, I placed him on the operating table, and made "Wilde's incision" on to the left mastoid process; but I found nothing here abnormal—no pus, and no serous exudation. I then endeavoured to open the mastoid antrum by making an opening in the bone with a small gouge; but after going as far as I felt justified in doing, I could find no cavity, and nothing to indicate any mischief here. The question now arose—What could be done further? The boy was evidently rapidly dying; and unless some relief could be quickly afforded, he would probably not live many days. The symptoms seemed to point to cerebral or cerebellar abscess, or to purulent meningitis.

I had had an opportunity of reading the three cases of trephining for cerebral abscess by Mr. Hulke, published in the *Lancet* for July 3rd, 1886, in one of which he readily reached an abscess in the cerebellum by an opening a little behind and below the mastoid process, and I determined to trephine here, hoping to reach the cerebellum just below the tentorium, immediately behind the angle where the lateral sinus passes down to the base of the skull. I made a crucial incision, and with a moderate sized trephine removed a piece of bone, exposing the dura mater. Then, with a scissors I made an incision in this membrane, intending to expose the surface of the cerebellum; but as I did so, a free gush of dark-coloured blood proved that I had wounded the lateral sinus at the upper angle of the wound. I at once inserted the point of my index finger into the opening, and with the slightest pressure completely arrested the hæmorrhage. I decided that nothing further could be done at that time; so I inserted a strip of lint, dusted with powdered iodoform, into the opening, and applied an antiseptic dressing, with a bandage round the head. The patient was then removed to bed, in exactly the same condition as before the operation.

From this time his symptoms underwent little or no alteration until his death, which took place on July 26th, four days after the operation. There was no further bleeding from the wounded lateral sinus. I removed the plug of lint on the second day, but as the wound was inclined to ooze I inserted another plug, which remained in up to his death. The temperature varied between 104° and 105° during the last few days, and the coma gradually increased.

At the *post-mortem* examination I found that the opening in the dura mater had opened the left lateral sinus about one inch behind the meatus, just at the angle which the sinus makes in that position as it turns downwards to the jugular foramen. Close to the wound in the sinus was a large thrombus, which almost completely obstructed the channel, and which was surrounded by a mass of thick pus, granulations, &c., lining the walls of the sinus. The thrombus occupied about an inch of the sinus, just in front of and a little above the trephine hole. I satisfied myself that it did not start from the wound in the sinus, but that it must have existed for some days previously. There was a little pus found superficial to the dura mater, just above the trephine hole; but this possibly was caused by the operation, although I could not be certain of it.

The brain was examined with the greatest care, and there was no sign whatever of an abscess in the cerebrum or in the cerebellum. There was no caries of the temporal bone, nor any meningitis on its surface.

The external auditory meatus, on the left side, was filled with succulent granulation tissue, which extended through the membrana tympani into the middle ear; this cavity also containing caseous and purulent material.

The mastoid cells were scarcely at all developed on the left side, which accounted for my difficulty in opening the mastoid antrum. The right ear was not examined.

Was it possible to diagnose during life that this was a case of phlebitis and not of cerebral abscess? Perhaps the high temperature and the somewhat rapid onset of the symptoms were points against abscess. In cases of cerebral abscess the temperature is often abnormally low, and the symptoms usually begin somewhat insidiously. Moreover, the pulse in cerebral abscess is usually slower than it was in this case.

The wound of the lateral sinus did no harm in this case, and it is well to know that hæmorrhage from these large venous trunks may be so readily arrested by pressure.

